# Opinions and use of neoadjuvant therapy for resectable, borderline resectable, and locally advanced pancreatic cancer: international survey and case-vignette study

**DOI:** 10.1186/s12885-019-5889-5

**Published:** 2019-07-09

**Authors:** Stefan Heinrich, Marc Besselink, Markus Moehler, Jean-Luc van Laethem, Michel Ducreux, Peter Grimminger, Jens Mittler, Hauke Lang, Manfred P. Lutz, Mickael Lesurtel, on behalf of the Scientific and Research Committee of the E-AHPBA and the EORTC pancreas working group

**Affiliations:** 1 Department of General, Visceral and Transplantation Surgery, University Hospital of Mainz, Langenbeckstrasse 1, 55131 Mainz, Germany; 2 Department of Surgery, Amsterdam UMC, Cancer Center Amsterdam, University of Amsterdam, Amsterdam, The Netherlands; 3 First Department of Internal Medicine, University Hospital of Mainz, Mainz, Germany; 4 Department of Gastroenterology and Digestive Oncology, Erasme Hospital, 1070 Brussels, Belgium; 5 Gastrointestinal Unit, Institute Gustave Roussy, Villejuif, France; 6 Department of Internal Medicine, CaritasKlinikum, Saarbrücken, Germany; 7 Department of Surgery and Liver Transplantation, Croix-Rousse University Hospital, University of Lyon, Lyon, France

**Keywords:** Pancreatic cancer, PDAC, Neoadjuvant therapy, Defintions, Resectability, Survey

## Abstract

**Background:**

Several new treatment options have become available for pancreatic ductal adenocarcinoma (PDAC), but the support for their use for resectable, borderline resectable and locally advanced PDAC is unclear.

**Methods:**

A survey was distributed to the members of the European-African Hepato-Pancreato Biliary Association (E-AHPBA) and the pancreas group of the European Organization for Research and Treatment of Cancer (EORTC) regarding 1) definitions of local resectability, 2) indications for neoadjuvant therapy and 3) case-vignettes regarding the resectability and treatment of PDAC.

**Results:**

In total, 114 participants from 37 countries were registered. About 35% of respondents, each, were of the opinion that borderline resectability is defined by any venous tumor contact and venous involvement < 180° or > 180°, respectively. The majority (75.4%) of participants believed that borderline resectable PDAC has a high risk for R1 resection and that neoadjuvant therapy might increase the R0-resection rate (79.8%) and improve oncological patient selection (84.2%). Chemotherapy was regarded useful to convert locally advanced to resectable PDAC by 55.7% of respondents. In the cases with resectable, borderline resectable, and locally advanced PDAC, 10 (8.8%), 78 (68.4%), 55 (48.2%) of participants would start with chemotherapy, respectively.

**Conclusions:**

Although definitions for borderline resectability differ among European surgeons, there seems to be a rather strong support for preoperative chemotherapy in PDAC aiming at minimizing R1 resections while increasing resection rates.

**Electronic supplementary material:**

The online version of this article (10.1186/s12885-019-5889-5) contains supplementary material, which is available to authorized users.

## Background

The treatment of pancreatic ductal adenocarcinoma (PDAC) has seen large changes during recent years. While surgery was only an option for resectable and non-metastatic PDAC in the past [1], modern poly-chemotherapeutic regimens (e.g. FOLFIRINOX or gemcitabine/nab-paclitaxel) with increased efficacy have changed the attitude to the management of PDAC: multimodality regimens are increasingly used to improve survival after curative resection, and increase resectability in both metastatic and non-metastatic disease [2–4]. In addition, the surgical understanding of a curative resection is changing: recent literature suggests that the long term outcome is markedly improved if the circumferential tumor free margins is at least 1 mm (R0 wide) [5].

Several expert panels and associations have established criteria for resectability of PDAC [6]. These definitions contain a subgroup of (borderline resectable) tumors, which may be considered resectable or unresectable based on the available surgical expertise. Most centers liberally offer venous resections despite an impaired long-term survival in deep venous infiltration (portal/superior mesenteric vein, pv/smv) and are highly restrictive with arterial resections in PDAC [7–9] because the limited oncological outcome adds to the risk for surgical morbidity. Consequently, the assessment of resectability of PDAC and the management of this subgroup varies widely based on the (surgical) expertise and the oncological practice of a center, but this heterogeneity complicates the interpretation of the current literature [2].

In parallel, neoadjuvant therapy is gaining popularity, but again indications may vary. Current literature suggests that a significant proportion of locally unresectable PDAC (=locally advanced PDAC, LAPC) may undergo radical resection with favourable outcome after adequate treatment response [10]. Recently, a randomized phase II-trial has reported a longer recurrence-free survival and R0-resection rate after neoadjuvant chemoradiation therapy for borderline resectable PDAC [11]. Even in primarily resectable tumors, resection margins appear wider, the proportion of lymph node metastases smaller, and survival superior following neoadjuvant chemotherapy. Since the definition and preoperative prediction of an R0 resection is difficult, any treatment with the potential aim of curative resection is considered neoadjuvant resulting in significant heterogeneity in the literature [2].

The aim of this survey was to analyze the current understanding of experts in the field regarding treatment aims and attitudes in the (neoadjuvant) management of pancreatic cancer.

## Methods

A web-based survey was distributed through the administrative offices of the European-African Hepato-Pancreato Biliary Association (E-AHPBA) and the pancreas group of the European Organization for Research and Treatment of Cancer (EORTC) to the respective members using Surveymonkey™. In addition to questions regarding the individual experience in the treatment of pancreatic cancer, the survey was designed to evaluate the attitude of European experts regarding the definition of local resectability and the indications for neoadjuvant therapy of PDAC of the pancreatic head.

Since participation to this European survey was anonymous and participation was voluntary and only offered to experts in the field, an approval of an ethical review board was not considered necessary.

### Survey

The SurveyMonkey™ platform was used. The survey contained 21 questions, of which 9 questions served to assess the individual experience of each participant in the management of PDAC, the understanding of definitions of resectability, and effects of neoadjuvant therapy on resectable, borderline resectable and unresectable PDAC (see Additional file 1).

#### Case vignettes

Four case vignettes were presented at the end of the survey by computed tomography images in order to assess the attitude of the participants regarding resectability and choice of treatment (see Additional file 2). Case 1 was a resectable, case 2 borderline and case 3 unresectable (locally advanced) PDAC. Lastly, case 4 was locally resectable with a solitary resectable liver metastasis. For each case, the participant had to assess resectability and to propose the optimal treatment. Furthermore, we asked about a potential aim of a neoadjuvant treatment in the particular case (3 questions per case).

### Statistics

All Analyses were performed using IBM SPSS 23 software. Categorial data between the groups were compared using the χ^2^-test. Differences were considered significant at a level of 0.05.

## Results

### Participants

In total, 114 participants were registered from 37 countries. Most of them originated from Europe, with most participants coming from Spain (*n* = 15), The Netherlands (*n* = 10), Great Britain (*n* = 9), Germany (*n* = 9) and Italy (*n* = 9). The vast majority of participants was hepato-pancreato-biliary (HPB) surgeons, and most of the participants reported an experience of more than 10 years in the management of patients with PDAC (Table 1). Amongst HPB and general surgeons, 52/84 (62%) and 9/15 (60%) reported an experience of more than 10 years in the management of PDAC, while only 8/84 (9.5%) and 3/15 (20%) had less than 5 years of experience, respectively. Similarly, 9/13 (69.2%) Gastrointestinal (GI) oncologists and all Radiation Oncologists had more than 10 years, while only 2 Medical Oncologists had less than 5 years of experience in managing PDAC patients.

**Table 1:** Participants characteristics

Participants	114
Experience in Treatment of PDAC	
< 5 years	13 (11.4%)
5–10 years	29 (25.4%)
> 10 years	72 (63.2%)
Scope of practice	
Surgery	99 (86.8%)
General surgery	84 (73.7%)
HPB surgery	15 (13.2%)
Oncology	15 (13.2%)
Medical oncology	12 (10.5%)
Radiation oncology	2 (1.8%)
Gastroenterology	1 (0.9%)
Origin of participants	
Europe	99 (86.8%)
Africa	8 (7%)
Middle East	5 (4.4%)
South America	2 (1.8%)
Countries	37

### Resectability

The participants had a high agreement (75.4%) that borderline resectability is associated with a high risk of R1 resections. In addition, a significant proportion of surgeons felt that borderline resectability inherits a higher surgical morbidity and requires a particular technical expertise (Table 2).

**Table 2:** Value of borderline resectability

	surgeons	oncologists
What does borderline resectable mean to you?		
The primary tumor can only be resected by surgeons with particular expertise	26/99 (26.3%)	2/15 (13.3%)
The resection of the primary tumor inherits a high risk for (incomplete) R1 resection	73/99 (73.7%)	13/15 (86.7%)
The tumor can be resected R0, but the oncological outcome after surgery is questionable	16/99 (16.9%)	1/15 (6.7%)
The morbidity of a resection of the primary tumor exceeds the normal morbidity by far	9/99 (9.1%)	–
Is not important – either a tumor is resectable or not	2/99 (2%)	–
What defines borderline resectability?		
Tumor contact to the portal (PV)/superior mesenteric (SMV) veins on imaging – likelihood of a PV/SMV resection	29/99 (29.3%)	8/15 (53.3%)
Tumor contact to the hepatic or mesenteric arteries on imaging	35/99 (35.4%)	4/15 (26.7%)
Tumor contact to the PV/SMV up to 180° on imaging	31/99 (31.3%)	6/15 (40%)
Tumor contact to celiac, hepatic or mesenteric arteries up to 180° on imaging	43/99 (43.4%)	5/15 (33.3%)
Tumor contact to the PV/SMV of more than 180° on preoperative imaging	40/99 (40.4%)	5/15 (33.3%)
Tumor contact to the celiac/hepatic or superior mesenteric arteries of more than 180° on imaging	19/99 (19.2%)	–
Tumor related portal vein thrombosis on imaging	16/99 (16.2%)	–
Resectability cannot be assessed on imaging only	12/99 (12.1%)	1/15 (6.7%)
others	9/99 9.1%)	–

While 39.5% of participants believed that any tumor contact > 180° defines borderline resectability, 32.5% each considered a venous contact up to 180° or any venous involvement as criteria for borderline resectability. Moreover, 42% of participants considered an arterial involvement up to 180°, and 34.2% any tumor contact to the superior mesenteric (sma) or hepatic artery as borderline resectable disease (Table 2).

The majority of participants (*n* = 74; 64.9%) considered locally unresectable tumors without metastases as locally advanced disease. Six participants did not fully agree with the offered definitions (Table 3).

**Table 3:** Definition of locally advanced disease

	Experience (years)			
	< 5 (*n* = 13)	5–10 (*n* = 29)	> 10 (*n* = 72)	total
Locally advanced disease describes a locally unresectable disease without evidence of metastases	11 (84.6%)	19 (65.6%)	44 (61.1%)	74 (64.9%)
Locally advanced disease is equvivalent to borderline resectability	–	5 (4.3%)	7 (6.1%)	12 (10.5%)
Locally advanced disease means a locally resectable disease with infiltration of mesenteric vascular structures	1 (7.7%)	3 (10.3%)	11 (15.3%)	15 (13.2%)
other	–	1 (8.8%)	5 (4.4%)	6 (5.3%)

### Treatment aims in cancer treatment

Being asked, which treatment aims are associated with palliative and adjuvant therapies in pancreatic cancer, 23 (20.2%) participants considered any postoperative treatment as adjuvant, all of which were surgeons - except one participant. Only eight participants believed that palliative treatment has a potential for patient cure, and about a third of participants (*n* = 37; 32.5%) was of the opinion that palliative therapy is associated with a prolongation of survival. Interestingly, the 80% of oncologists (12/15) associated a palliative treatment with a prolongation of survival, while on a 25.3% of surgeons (25/99) had the same association. Moreover, (86.7%) of oncologists (13/15) and 63.6% of surgeons (63/99) were of the opinion that the aim of a palliative therapy is to relief symptoms. The vast majority of participants, however, was of the opinion that the aim of adjuvant therapy is to reduce the recurrence risk after complete tumor resection (92/114; 80.7%), while palliative treatments should relief symptoms (75/114; 65.8%).

Considering neoadjuvant therapy, 91 participants (79.8%) replied that neoadjuvant therapy has the aim to increase the R0-resection rate in borderline resectable cancer. Also, 45 (39.5%) respondents considered the treatment of micrometastases and 33 (28.9%) a decreased risk of metastases in resectable cancer as treatment aims of neoadjuvant therapy. Moreover, 31 (27.2%) participants believed that the aim of neoadjuvant therapy is to increase the size of the resection margin in resectable as well as borderline resectable cancer (Table 4). On the other hand, 64 (55.3%) respondents were of the opinion that the aim of neoadjuvant therapy can be the convertion of LAPC to resectable disease or even to stabilize oligo-metastatic disease with the aim of a secondary surgical treatment (*n* = 17; 14.9%).

**Table 4:** Treatment aims/advantages of neoadjuvant therapy

	surgeons	oncologists
Which treatment aims do you associate with neoadjuvant therapy for PDAC?		
increasing the size of the resection margin (in resectable or borderline resectable cancer)	27/99 (27.3%)	4/15 (26.7%)
decreasing the risk of distant metastases after an apparently curative resection by a preoperative treatment	27/99 (27.3%)	6/15 (40%)
increasing the R0 resection rate (e.g. in borderline resectable cancer)	77/99 (77.8%)	14/15 (93.3%)
achieving resectability/disease stabilization in oligometastasized disease with the aim of surgical treatment	16/99 (16.2%)	2/15 (13.3%)
achieving secondary resectability in locally unresectable disease	54/99 (54.5%)	9/15 (60%)
Preoperative treatment of micrometastases	35/99 (35.4%)	10/15 (66.7%)
What are the theoretical advantages of neoadjuvant over adjuvant treatment?		
better treatment tolerability of neoadjuvant treatment	41/99 (41.4%)	12/15 (80%)
higher dosage possible during neoadjuvant treatment	26/99 (26.3%)	7/15 (46.7%)
lower surgical complication rate after neoadjuvant treatment	11/99 (11.1%)	3/15 (20%)
better oncological patient selection by neoadjuvant treatment	83/99 (83.8%)	13/15 (86.7%)
better vascular supply of the tumor for neoadjuvant treatment	24/99 (24.2%)	6/15 (40%)

In summary, 39.5% of the respondents associated neoadjuvant therapy with the treatment of micrometastases. Moreover, more participants (79.8%) considered neoadjuvant treatment to increase the R0-resection rate of borderline resectable PDAC than to convert unresectable to resectable PDAC (55.3%). We also found a tendency that oncologists more often believe that the treatment of micrometastases is the primary aim of neoadjuvant therapy. Since multiple answers were allowed to this question, we found a large overlap between the replies.

### Neoadjuvant vs adjuvant therapy

The majority of participants (96/114, 84.2%) considered the oncological patient selection as the strongest advantage of neoadjuvant therapy over adjuvant therapy, meaning that surgery would be avoided in patients with progressive disease. A quarter of respondents believed that the blood supply to the tumor is better during neoadjuvant than adjuvant therapy. Only 12.3% (*n* = 14) of respondents were of the opinion that the surgical morbidity is lower after neoadjuvant therapy. Nearly half of the participants felt that neoadjuvant therapy was better tolerated than adjuvant therapy. However, these differences did not reach statistical significance.

### Case vignettes

#### Resectability

The assessment of resectability of the resectable, borderline resectable and locally unresectable tumors matched with the intention of the survey and findings on the CT images in the majority of cases. Most of the participants (84.2%) considered the resectable tumor as resectable, while three were not sure and five participants were of the opinion that the tumor was borderline resectable.

Similarly, most participants judged the borderline resectable tumor as true borderline resectable (*n* = 79; 69.3%), 11 respondents considered the tumor as upfront resectable, and 10 as unresectable. Only two were not sure.

**Fig. 1:**
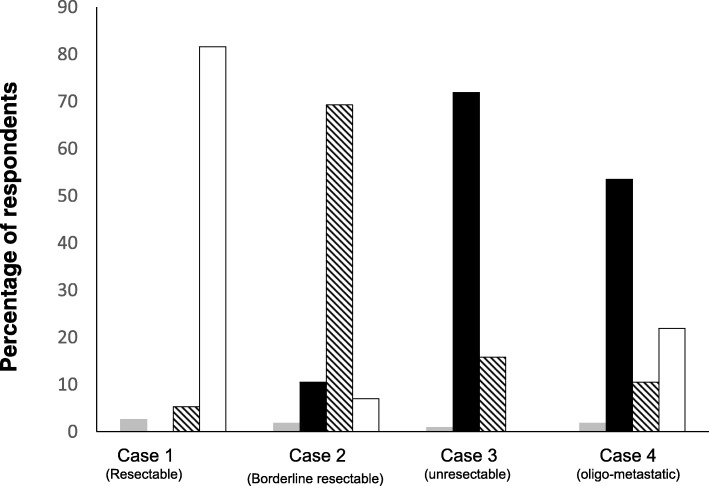
Assessment of the clinical cases regarding resectability of the disease (not sure,  unresectable,  borderline resectable,  resectable)

The unresectable tumor was considered unresectable by 72% of the participants, while 16% had the impression the tumor was borderline resectable. None of the participants thought this was a resectable tumor, and one was not sure (Fig. 1).

#### Treatment including neoadjuvant therapy

**Fig. 2:**
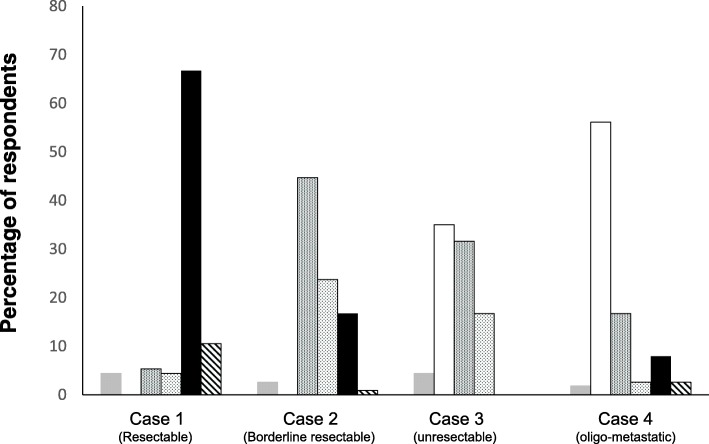
Proposition of the treatment for the different clinical scenarios ( others,  neoadjuvant chemo-radiotherapy,  neoadjuvant chemotherapy,  palliative chemotherapy,  Surgery + adj. Chemotherapy,  surgery only)

**Fig. 3:**
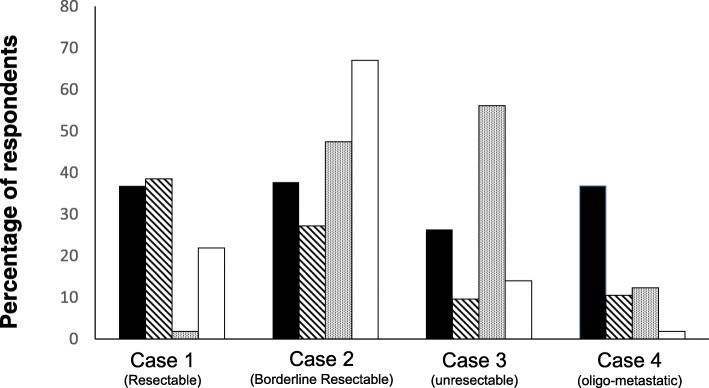
Treatment aims for the four clinical cases suggested by the respondents ( improving the long-term survival,  decrease risk of metastasis,  achieve resectability,  increase the probability of R0 resection)

The majority of participants would treat primarily resectable PDAC (case 1) with upfront surgery followed by adjuvant chemotherapy (65%). However, 11% recommended surgery-only (without adjuvant therapy), and 9% would start with a neoadjuvant therapy (6 chemotherapy, 4 chemo-radiotherapy) (Fig. 2). In this case, 45 participants (39.5%) defined a potential aim of neoadjuvant therapy as a decreased risk of tumor recurrence (metastases) and 42 (36.8%) to improve long-term survival. In addition, 25 (21.9%) participants would apply neoadjuvant therapy to increase the probability of R0 resection, while only two would apply it to achieve (secondary) resectability (Fig. 3).

In the borderline resectable tumor *(case 2)*, about half of the respondents (48%) would start with a neoadjuvant treatment, of which 15 (13.2%) would treat the patient with chemotherapy, and 27 (23.7%) with chemoradiotherapy. Only 20 participants (17.5%) would primarily explore the patient with the aim of a primary resection, and 19 of them would also give adjuvant therapy after an apparently curative resection (Fig. 2). The rationale for a neoadjuvant therapy in such borderline resectable tumors was to achieve resectability by 54 (47%) and to increase the rate of R0-resections by 72 (63%) participants. Again, 27% participants considered a decreased risk of metastasis and 38% an improvement of long-term survival as potential aims of neoadjuvant therapy in borderline resectable cancer (Fig. 3).

In the locally unresectable tumor *(case 3)*, about half of participants (*n* = 55, 48%) would start with a neoadjuvant therapy with consequent surgery in case of adequate tumor response, while 41 (36%) would apply palliative chemotherapy. As neoadjuvant treatment, chemotherapy was preferred (*n* = 36, 31.6%) over chemo-radiotherapy (*n* = 19, 16.7%), and five participants would use other treatments (Fig. 2). The majority of respondents (55%) was of the opinion that the aim of neoadjuvant therapy in unresectable cancer is to achieve resectability, and a fourth of participants considered an improvement in long-term survival as the primary treatment aim. Only 16 and 11 participants, respectively, considered an increase in the R0-resection rate and a decreased risk of metastases as treatment aims in this particular situation (Fig. 3).

Lastly, 54% of the participants considered a locally resectable pancreatic cancer with a solitary (resectable) liver metastasis *(case 4)* as unresectable (≈non-surgical) disease. In contrast, 26 respondants (22.8%) considered this clinical scenario as primarily resectable, and 12 as borderline resectable disease. Only two participants were not sure (Fig. 1). Consequently, 56% recommended palliative chemotherapy, while 17% would apply a neoadjuvant chemotherapy. Twelve participants (10.5%) proposed upfront surgery, of whom 9 would apply adjuvant chemotherapy thereafter. Three participants would start with neoadjuvant chemoradiotherapy and two with another treatment (Fig. 2). The majority (38%) proposed a neoadjuvant therapy in order to increase long-term survival (Fig. 3).

## Discussion

This first pan-European survey performed by E-AHPBA and EORTC on neoadjuvant treatment in pancreatic cancer found that the definition of borderline resectability (and unresectability) varies among European experts. These different definitions often do not affect the clinical management of patients with PDAC. This study confirms a paradigm shift in the understanding and treatment of patients with locally advanced and oligo-metastatic cancer, but also a broad heterogeneity in the management of such patients. For resectable PDAC, primary resection followed by adjuvant chemotherapy was the preferred concept, and a neoadjuvant approach was only recommended by a few participants. About half of the respondents would initiate neoadjuvant therapy in borderline resectable and in LAPC. Finally, neoadjuvant therapy was only recommended by a minority of participants for metastatic PDAC.

Following most classifications, a resectable tumor has a maximal tumor contact to the pv/smv of less than 180° without any contact to the celiac or superior mesenteric artery. Borderline resectability includes tumors which have more than 180° contact to the pv/smv and up to 180° contact to the celiac/superior mesenteric arteries. Tumor infiltration of the celiac/superior mesenteric arteries beyond 180° defines unresectable disease [6]. Although differences in the assessment of resectability are generally attributed to the surgical expertise of centers in the literature, this survey demonstrates that such differences are more likely related to different perceptions of PDAC disease and different oncological attitudes considering the experience in this field of most participating surgeons: while some may primarily consider the technical success of surgery, others predominantly see the questionable oncological benefit of an R1 resection. Accordingly, resectability rates in series on neoadjuvant therapy depend on the inclusion policy in addition to the surgical expertise and the applied treatment.

The heterogeneity in treatment concepts for unresectable or oligo-metastatic cancer is even more pronounced and mainly reflects the controversial literature: a few recent publications suggest favorable long-term outcome of patients with unresectable or even oligo-metastatic disease after response to FOLFIRINOX [10, 12, 13]. Although these are retrospective studies, some centers have already adopted these concepts for selected patients. Thus, a large proportion of participants considered both scenarios as potentially curable in a multimodality concept.

In contrast to well established definitions of adjuvant and palliative treatment aims, a clear definition of treatment aims for a neoadjuvant treatment has not been established, yet. Accordingly, about half of the recipients considered the conversion from an unresectable to a resectable disease as aim of neoadjuvant therapy, while ¾ of them considered the increase in the R0-resection rate as aim of neoadjuvant therapy. Considering the aim of an adjuvant treatment (reduction of tumor recurrence) and the homology of the terms “adjuvant” and “neoadjuvant”, the primary aim of a *neoadjuvant treatment* should be to decrease the recurrence risk after a curative resection, which may be attributed to fewer lymph node metastases as well as the treatment of micrometastases and circulating tumor cells as indicated by most participants. Further beneficial effects may be larger resection margins, a lower R1 resection rate and smaller tumors. In locally unresectable tumors or whenever the risk of an R1 (wide) resection is high, the primary treatment aim of a preoperative therapy is to shrink the tumor and convert a (potentially) unresectable to a resectable disease. In this scenario, the treatment should be considered a *down-sizing* or *conversion therapy* [2]. If secondary resectability is not attempted (or unprobable), a palliative therapy is indicated. Similarly, neoadjuvant therapy for other tumors such as breast or upper GI cancer implies the preoperative therapy of technically resectable tumors with the aim of a reduction of tumor recurrence [14–16]. Also for colorectal liver metastases, many authors differentiate a “conversion” and a “neoadjuvant” chemotherapy [17–19].

Such differentiation in the terminology of treatment concepts would particularly increase the comparability of results in the borderline resectable group: a borderline resectable tumor (e.g. infiltration of the smv) according to current definitions could be treated by a neoadjuvant therapy with an expected high resectability rate – since it is most probably primarily resectable and would be resected upfront in many experienced centers. Since a deep infiltration may be associated with impaired outcome [9], a real *neoadjuvant therapy* could be given with the aim of an improvement of recurrence-free and overall survival in this scenario. The same idea is the basis of *neoadjuvant therapy* in resectable tumors [6]. A primarily unresectable disease, however, would undergo *conversion therapy* with an estimated lower secondary resection rate (depending on the tumor response rate). The same would be true for a borderline resectable tumor with tumor contact to the superior mesenteric artery – a scenario with a high risk of R1- or even un-resectability. Moreover, in the case of a resectable primary tumor with resectable liver metastases, the aim of a *neoadjuvant therapy* would be to decrease recurrence rate (e.g. new metastases) after the resection of both the primary tumor and metastases (e.g. case 4).

These two separate definitions of “neoadjuvant” treatment would not necessarily change the management of the patient, since the same treatment is often applied in both situations. However, they might help to better define inclusion criteria for clinical studies and the reading of the literature. If a high response rate is required for a locally unresectable tumor, the conversion therapy should be a maximally potent local treatment (including chemo-radiation), while a systemic treatment could be more suitable to prevent metastatic recurrence in some borderline resectable tumors (e.g. portal vein infiltration).

## Conclusions

In conclusion, this first E-AHPBA/EORTC pan-European survey documents a shift in attitudes regarding the management of advanced PDAC among HPB surgeons and oncologists with fairly large support for neoadjuvant treatment. Moreover, this survey depicts different understandings of borderline resectability and neoadjuvant therapy which should trigger a discussion on an adaption of definitions in the setting of PDAC.

## Additional files

Additional file 1: Questions of the survery (number of potential answers indicated). (PDF 323 kb)

Additional file 2: Case vignettes (provided including CT scans). (PDF 362 kb)

## Data Availability

The datasets used and/or analysed during the current study available from the corresponding author on reasonable request.

## Abbreviations

E-AHPBA: European-African Hepato-Pancreato Biliary Association
EORTC: European Organization for Research and Treatment of Cancer
GI: Gastrointestinal
HPB: Hepato-pancreato-biliary
LAPC: Locally advanced pancreatic cancer
PDAC: Pancreatic ductal adenocarcinoma
pv: Portal vein
sma: Superior mesenteric artery
smv: Superior mesenteric vein
