# Supplementary material for: Opinions and use of neoadjuvant therapy for resectable, borderline resectable, and locally advanced pancreatic cancer: international survey and case-vignette study
Source: BMC Cancer. 2019 Jul 9;19:675. doi: 10.1186/s12885-019-5889-5 (PMC6617881; doi:10.1186/s12885-019-5889-5)
Supplement: Supplementary file 1 — Questions of the survery (number of potential answers indicated). (PDF 323 kb) [file 12885_2019_5889_MOESM1_ESM.pdf]

Supplementary File 1

Questions of the survey

Q1 What is your speciality? (single answer)

- Medical Oncology
- Radiation Oncology
- General Surgery
- HPB surgery
- Other (please specify)

Q2 Through which association did you receive this survey? (single answer)

- E-AHPBA
- EORTC

Q3 For how long have you been treating patients with pancreatic cancer? (single answer)

- <5years
- 5-10years
- >10years

Q4 What does *bordeline resectable* mean to you? (multiple answers possible)

- The primary tumor can only be resected by surgeons with particular expertise
- The resection of the primary tumor inherits a high risk for (incomplete) R1 resection
- The tumor can be resected R0, but the oncological outcome after surgery is questionable
- The morbidity of a resection of the primary tumor exceeds the normal morbidity by far
- Is not important – either a tumor is resectable or not

Q5 What defines *borderline resectability* in your opinion? (multiple answers possible)

- Tumor contact to the portal (PV)/superior mesenteric (SMV) veins on imaging – likelihood of a PV/SMV resection
- Tumor contact to the hepatic or mesenteric arteries on imaging
- Tumor contact to the PV/SMV up to 180° on imaging
- Tumor contact to celiac, hepatic or mesenteric arteries up to 180° on imaging
- Tumor contact to the PV/SMV of more than 180° on preoperative imaging
- Tumor contact to the celiac/hepatic or superior mesenteric arteries of more than 180° on imaging
- Tumor related portal vein thrombosis on imaging
- Resectability cannot be assessed on imaging only
- Other (please specify)

Q6 What defines *locally advanced disease* in your opinion? (multiple answers possible)

- Locally advanced disease is equvivalent to borderline resectability
- Locally advanced disease means a locally resectable disease with infiltration of mesenteric vascular structures
- Locally advanced disease describes a locally unresectable disease without evidence of metastases

Q7 Which of the following treatment aims hold true for patients with pancreatic cancer? (multiple answers possible)

- palliative treatment – relief of symptoms
- palliative treatment – patient cure possible
- adjuvant treatment – reducing the risk of disease recurrence after complete tumor resection
- adjuvant treatment – any treatment after surgery

- palliative treatment – prolongation of patient survival

Q8 Which treatment aims do you associate with *neoadjuvant therapy* for pancreatic cancer? (multiple answers possible)

- preoperative treatment of micrometastases
- achieving secondary resectability in locally unresectable disease
- achieving resectability/disease stabilization in oligometastasized disease with the aim of surgical treatment
- increasing the R0 resection rate (e.g. in borderline resectable cancer)
- decreasing the risk of distant metastases after an apparently curative resection by a preoperative Treatment
- increasing the size of the resection margin (in resectable or borderline resectable cancer)

Q9 What are the theoretical advantages of neoadjuvant over adjuvant treatment? (multiple answers possible)

- better treatment tolerability of neoadjuvant treatment
- higher dosage possible during neoadjuvant treatment
- lower surgical complication rate after neoadjuvant treatment
- better oncological patient selection by neoadjuvant treatment (patients with progressive disease under neoadjuvant treatment are excluded)
- better vascular supply of the tumor for neoadjuvant treatment
