# Supplementary material for: Opinions and use of neoadjuvant therapy for resectable, borderline resectable, and locally advanced pancreatic cancer: international survey and case-vignette study
Source: BMC Cancer. 2019 Jul 9;19:675. doi: 10.1186/s12885-019-5889-5 (PMC6617881; doi:10.1186/s12885-019-5889-5)
Supplement: Supplementary file 2 — Case vignettes (provided including CT scans). (PDF 362 kb) [file 12885_2019_5889_MOESM2_ESM.pdf]

Supplementary File 2

Case vignettes

Case #1

The CT scan describes the maximal extension of disease (Tumor marked and measured; arrow: superior mesenteric vein; no other significant findings)

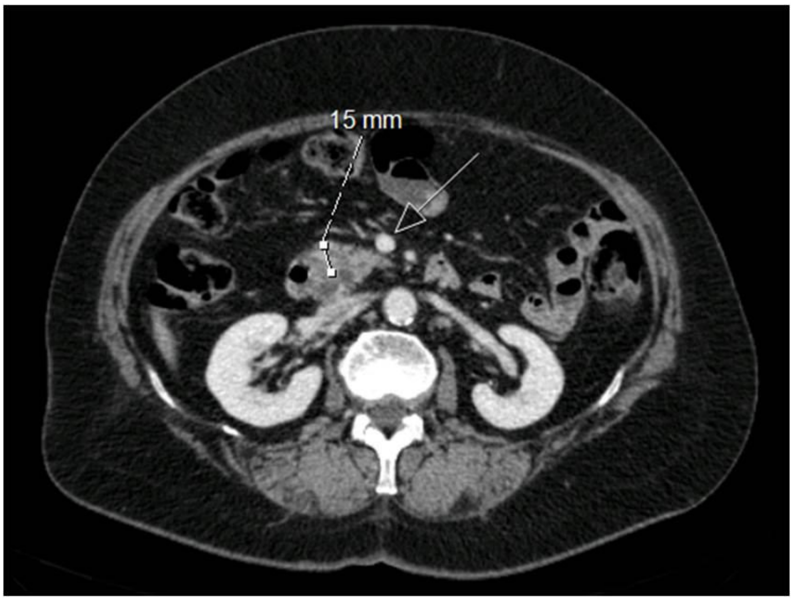

Q10: The tumor is (single answer)

- o resectable
- o borderline resectable
- o unresectable
- o I am not sure

Q11: Which treatment would you recommend? (single answer)

- o Neoadjuvant chemotherapy
- o Neoadjuvant Chemo-radiotherapy
- o Surgery only
- o Surgery + adjuvant chemotherapy
- o Palliative chemotherapy
- o Other (please specify)

Q12: What would be the rationale for a neoadjuvant therapy <u>in this case</u>? (multiple answers possible)

- o Increasing the probability of R0 resection
- o Achieving resectability
- o Decreasing the risk of metastasis after curative resection
- o Improving long-term survival
- o Other (please specify)

Case #2

On this CT scan the tumor has contact to the superior mesenteric **vein** (arrow). The CT scan describes the maximal extension of disease (no other significant findings)

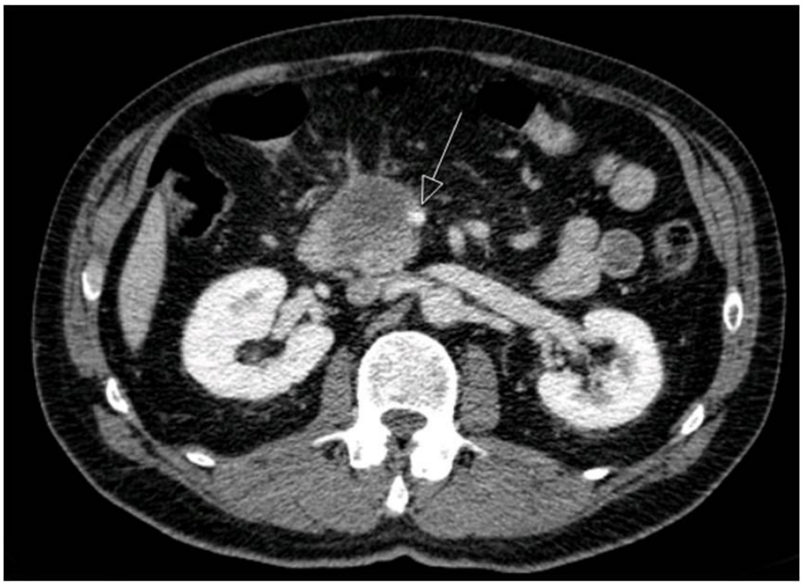

The coronal reconstruction of the above CT scan reveals the localization of the tumor contact to the superior mesenteric **vein** (arrow).

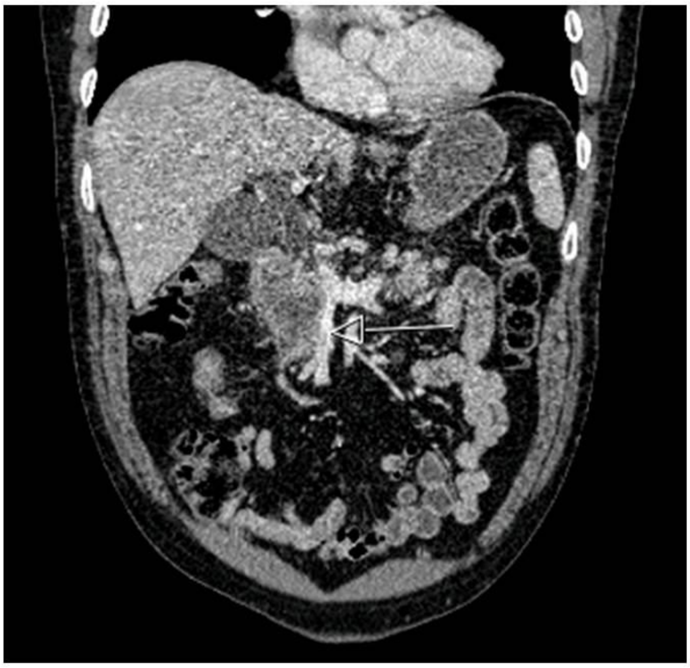

Q13: The tumor is (single answer)

- resectable
- borderline resectable
- unresectable
- I am not sure

Q14: Which treatment would you recommend? (single answer)

- Neoadjuvant chemotherapy
- Neoadjuvant Chemo-radiotherapy
- Surgery only
- Surgery + adjuvant chemotherapy
- Palliative chemotherapy
- Other (please specify)

Q15: What would be the rationale for a neoadjuvant therapy <u>in this case</u>? (multiple answers possible)

- Increasing the probability of R0 resection
- Achieving resectability
- Decreasing the risk of metastasis after curative resection
- Improving long-term survival
- Other (please specify)

Case #3

This CT scan describes the maximal extension of disease (Arrow: superior mesenteric artery; no other significant findings)

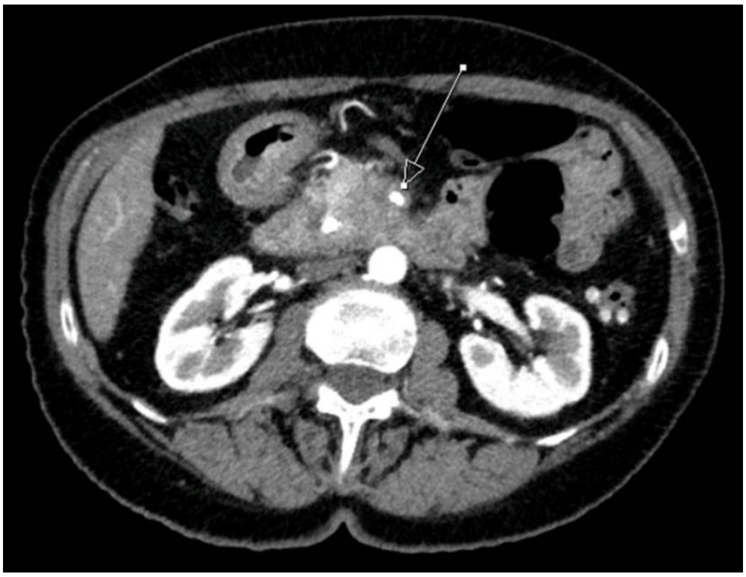

Q16: The tumor is (single answer)

- resectable
- borderline resectable
- unresectable
- I am not sure

Q17: Which treatment would you recommend? (single answer)

- Neoadjuvant chemotherapy
- Neoadjuvant Chemo-radiotherapy
- Surgery only
- Surgery + adjuvant chemotherapy
- Palliative chemotherapy
- Other (please specify)

Q18: What would be the rationale for a neoadjuvant therapy <u>in this case</u>? (multiple answers possible)

- Increasing the probability of R0 resection
- Achieving resectability
- Decreasing the risk of metastasis after curative resection
- Improving long-term survival
- Other (please specify)

Case #4

This CT scan reveals a singular liver metastasis (arrow). The CT scan describes the maximal extension of disease (no other significant findings)

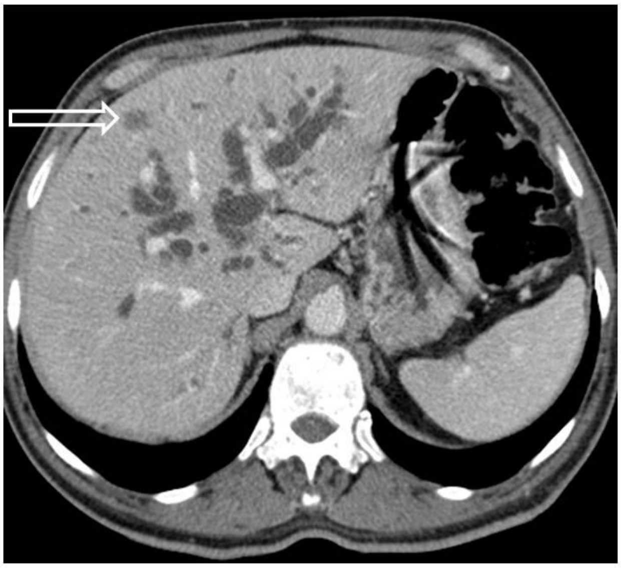

Q19: The tumor is (single answer)

- o resectable
- o borderline resectable
- o unresectable
- o I am not sure

Q20: Which treatment would you recommend?

- o Neoadjuvant chemotherapy
- o Neoadjuvant Chemo-radiotherapy
- o Surgery only
- o Surgery + adjuvant chemotherapy
- o Palliative chemotherapy
- o Other (please specify)

Q21: What would be the rationale for a neoadjuvant therapy <u>in this case</u>? (multiple answers possible)

- o Increasing the probability of R0 resection
- o Achieving resectability
- o Decreasing the risk of metastasis after curative resection
- o Improving long-term survival
- o Other (please specify)
